# The Effect of Internal Gas Pressure on the Compression Properties of Natural Rubber Foams

**DOI:** 10.3390/ma17081860

**Published:** 2024-04-18

**Authors:** Amirhosein Heydari, Denis Rodrigue

**Affiliations:** Department of Chemical Engineering, Laval University, Quebec, QC G1V 0A6, Canada; amirhosein.heydari.1@ulaval.ca

**Keywords:** natural rubber, foams, finite element method, hyper-elastic model, internal gas pressure

## Abstract

This study explores the effect of internal gas pressure (P) on closed-cell natural rubber (NR) foams. Three key factors are analyzed using a 3D model during uniaxial compression: (1) the initial gas pressure (P_0_ = 1, 2, and 3 atm) inside the cells, (2) different cell sizes (D = 0.1, 0.2, 0.3, and 0.4 mm in diameter), and (3) the presence of defects (holes in the cell walls) in terms of their sizes (d = 0.07 to 0.1 mm). The findings reveal a negative relationship between the initial gas pressure and the relative internal gas pressure (α = P/P_0_) and a direct correlation with stress during compression. For instance, a change from 1 to 3 atm of the initial internal gas pressure results in a 158% decrease in α with only a 3% increase in stress. Larger cell sizes contribute to higher α but lower stress levels during compression. Changing the cell size from 0.1 to 0.4 mm generates a 27% increase in α but a 45% drop in stress. An analysis of hole sizes (cell connection) indicates that larger holes result in higher relative internal gas pressure, while smaller holes lead to higher stress levels because of more flow restriction. For example, increasing the hole size from 0.07 to 0.1 mm leads to an 8% higher α but a 32% stress reduction. These findings highlight the significant effect of the internal gas pressure inside the cells in determining the mechanical properties of rubber foams, which are generally neglected. The results also provide useful insights for better material design and different industrial applications. This study also generates predictive models to understand the relationships between stress, strain, initial gas pressure, cell size, and defects (holes/connections), enabling the production of tailor-made rubber foams by controlling their mechanical behavior.

## 1. Introduction

Natural rubber (NR) foams, known for their flexibility and resilience, have a broad range of applications across different industries. They provide comfort and sound insulation in the automotive sector, contribute to thermal and acoustic insulation in construction, and can be found in everyday items such as shoe soles, sports equipment, and packaging materials [1,2,3,4]. As for any porous material, NR foams can be divided into two types: open cells, which have interconnected cells throughout the matrix and open to the ambient environment, and closed cells, where the gas is dispersed separately, and individual cells throughout the matrix are isolated from the outside [5,6,7].

Researchers have long recognized the significance of natural rubber and have devoted considerable efforts to understanding their mechanical properties [8,9]. However, relatively limited research has focused on closed-cell NR foams. The main challenge to understanding the mechanical properties of these foams lies in the presence of the trapped gas inside the cells and their interaction with the foam structure/morphology. Consequently, gaining a deeper insight into the mechanical behavior of closed-cell foams requires a comprehensive analysis of how these cells behave when the gas phase is confined under different deformation (compression, tension, and shear).

During the manufacturing process, the trapped gas molecules inside the cells exert pressure on the surrounding matrix. This pressure will influence the shape, orientation, distribution, and size of the cells, having a direct effect on the mechanical properties of the foams [10]. The effect of internal gas pressure on the mechanical behavior of closed-cell foams was first reported by Shaw and Sata in 1966 while studying polystyrene (PS) foams [11]. The studies then focused on the effect of internal gas pressure in closed-cell foams, especially those with high porosity [10,12,13,14,15,16,17,18]. Even in foams with low porosity, the effect of internal gas pressure can be significant, affecting the performance and stability of the final materials. For instance, elastomeric seals may lose their stability due to cavitation resulting from rapid depressurization in high-pressure gas tanks, which is attributed to internal gas pressure effects [19]. Therefore, understanding the relationships between the internal gas pressure and mechanical behavior of closed-cell foams is of high interest to optimize the properties of both high- and low-density foams.

Gibson and Ashby studied the effect of internal gas pressure [16,20]. They presented a semi-empirical model to determine Young’s modulus of porous polymeric materials under isothermal conditions. The model assumes that the cells have a polygonal structure, and the foam modulus is composed of three parts (contributions): the cells’ edges, the cells’ surface, and the internal gas pressure inside the cells leading to:

(1) $$E_{f}=E_{edge}+E_{surface}+E_{gas}=\phi^{2}{\left(\frac{\rho_{f}}{\rho_{m}}\right)}^{2}+\left(1-\phi \right)\left(\frac{\rho_{f}}{\rho_{m}}\right)+E_{gas}$$

where *E_f_* is the Young’s modulus of the foam, $E_{edge}$ is the Young’s modulus of the edges, $E_{gas}$ is the Young’s modulus of the gas, and $E_{surface}$ is the Young’s modulus of the cell surface, while $\rho_{f}$ is the density of the foam, $\rho_{m}$ is the density of the matrix, and *ϕ* is the relative amount of matrix located in the corners of the cells.

Assuming that the gas inside the cell is ideal [16], the gas contribution can be calculated as:

(2) $$E_{gas}=\frac{d(\Delta P)}{d\varepsilon }=\frac{1-2\vartheta }{1-R}P_{0}$$

where *P*_0_ is the initial gas pressure inside the cell, *R* (= $\frac{\rho_{f}}{\rho_{m}}$) is the relative density, and *ʋ* is the Poisson ratio. By substituting Equation (2) into Equation (1), we can determine the elastic modulus of the foam:

(3) $$\frac{E_{f}}{E_{m}}=\phi^{2}R^{2}+\left(1-\phi \right)R+\frac{P_{0}}{E_{m}}\frac{1-2\vartheta }{1-R}$$

In most cases studied in the literature, the initial gas pressure (*P*_0_) is very low compared to the matrix modulus (*E_m_*); therefore, the last term of Equation (3) can be disregarded, resulting in a simplified form:

(4) $$\frac{E_{f}}{E_{m}}\;≅\;\phi^{2}\;{\left(R\right)}^{2}+(1-\phi )R$$

However, this is not always the case, especially for elastomeric foams where the unfoamed modulus (*E_m_*) can be quite low.

As for several engineering problems, three main scientific approaches can be used to study the effect of any parameter: (1) analytical analysis, (2) experimental analysis, and (3) numerical analysis. However, the most effective studies often combine these approaches to achieve a complete understanding with validation.

For the analytical analysis of the internal gas pressure, Kitazono and colleagues determined the mechanical properties of porous materials, including Young’s modulus, Poisson’s ratio, and yield stress, using a mean field approximation and equivalent inclusion methods [21]. They assumed that the internal gas pressure had a negligible effect on the mechanical properties of metal foams due to their very high moduli. Using a micromechanical second-order moment model for the stress, Zhang et al. [22] achieved more accurate calculations of the mechanical behavior of porous materials under different internal gas pressures. Contrary to Kitazono’s claims [21], their results revealed a significant effect of internal gas pressure on reduced yield strain [22].

For experimental analysis at the micro-mechanical level, the direct measurement of internal gas pressure by experimental methods can be very challenging, time-consuming, and expensive, if not impossible. While indirect analysis of the effect of internal gas pressure on the mechanical properties of cellular materials has been conducted [13,14,15,23], some researchers used innovative experimental approaches to directly and indirectly measure the internal cellular pressure in porous materials. For example, Bouix designed an axial compression test for polypropylene (PP) foams inside a water chamber and controlled the amount of air bubbles leaking from the foam during compression by varying the strain rate [12]. Zhang and Yu investigated the effect of internal gas pressure on the strength and deformation of pipes [24]. They used a pressurized, thin-walled circular tube and reported their findings based on two mechanisms: the direct effect of internal gas pressure and the indirect effect caused by the interaction between the internal gas pressure and the solid wall of the tube. Xu et al. [25] examined the effect of internal gas pressure on the strength of aluminum (Al) foams with regular shapes and different pore content at their sealed ends. They put honeycomb materials between two fiber-reinforced sheets, and they made some holes in another sheet. First, they looked at how the air escaped from these holes with different contents. Then, the strain rate sensitivity was studied by a series of compression tests. In summary, researchers have overcome challenges in directly measuring micro-mechanical internal gas pressure by using innovative experimental methods. Although simplifications have been made in these experimental tests, their creativity helped us to learn more about how internal gas pressure affects the strength of materials, making important progress in our knowledge.

Previous numerical analyses investigating the effect of internal gas pressure in foams can be divided into two main categories: studies based on two-dimensional (2D) finite element analysis (FEA) and studies based on three-dimensional (3D) FEA. In the field of 2D analysis of porous materials, Hönig and Ruan investigated the mechanical behavior using 2D honeycomb models combined with experimental evidence for this honeycomb structure [26,27]. Among all the work performed, the studies by Ma [28], Liu [29], and Ozgur [30], as well as Sun and Li [23], are interesting as they tried to simulate the mechanical behavior of porous materials with the help of 2D finite element methods (FEMs). It is important to note that for all these studies, the results obtained did not quite match the experimental ones because they oversimplified the problem from its real 3D geometry to a simpler 2D one.

With improvements in the processing power of computers over the years, researchers were inclined toward three-dimensional (3D) calculations. In 2004, Öchsner and Mishuris studied the mechanical properties of porous materials with a 3D representation of a simple cubic unit cell model and accounted for the effect of internal gas pressure [31]. In 2010, Xu [32] investigated the mechanical behavior of closed-cell metal (aluminum alloy) foams with internal cell pressure using a 3D face center cubic (FCC) and body center cubic (BCC) model. In 2015, Fang analyzed closed-cell metal foams with 3D numerical simulations and used a general mesoscopic model including several random cells [33]. Heydari et al. [34] studied the 3D geometric modeling of NR foams by using scanning electron microscope (SEM) images to build their structure for FEMs and investigated the effect of several factors, such as the relative foam density, on the mechanical properties. But recently, with computers becoming increasingly powerful, researchers succeeded in moving from 2D calculations to 3D ones and performed analyses that are closer to the real morphology of the foam leading to more realistic results.

Based on the current literature, it is clear that a gap in research still exists regarding the effect of internal gas pressure on the mechanical properties of porous polymers, especially rubber foams. Therefore, more specific investigations are needed in this field. For this purpose, this work aims to explore the effect of internal gas pressure on the mechanical behavior of closed-cell natural rubber foams using a finite element 3D model. The investigation focuses on three main aspects: (1) assessing the effect of different initial gas pressure (P_0_ = 1, 2, and 3 atm) inside the cells, (2) investigating the effect of internal gas pressure with different cell sizes (D = 0.1, 0.2, 0.3, and 0.4 mm), and (3) analyzing at the micro level the presence of defects (connections/holes) in the closed cells structure by adding different hole sizes (d = 0.07, 0.08, 0.09, and 0.1 mm) to investigate partially open cells.

## 2. Numerical Simulations

### 2.1. Geometry and Material Definition

The 3D cell structure was obtained from the foam morphology (average cell size), and the element was defined based on a cubic representative volume element (RVE) with a central cell, as described in our previous articles [8,34]. ANSYS workbench 2022 R1 software was used for the material definition and finite element analysis. The Model Mooney-Rivlin 5 parameters hyper-elastic model was selected to simulate the mechanical behavior of NR, as discussed in our previous papers [8,34]. The model is presented in Equation (5), while the values of the coefficients are reported in Table 1.

U = C_10_ (I_1_ − 3) + C_01_ (I_2_ − 3) + C_20_ (I_1_ − 3)^2^ + C_11_ (I_1_ − 3) (I_2_ − 3) + C_02_ (I_2_ − 3)^2^ (5)

where U is the strain energy density function, I_1_ and I_2_ are the first and second strain invariants, respectively, and *C_ij_* is the material constant, as reported in Table 1.

**Figure 1:**
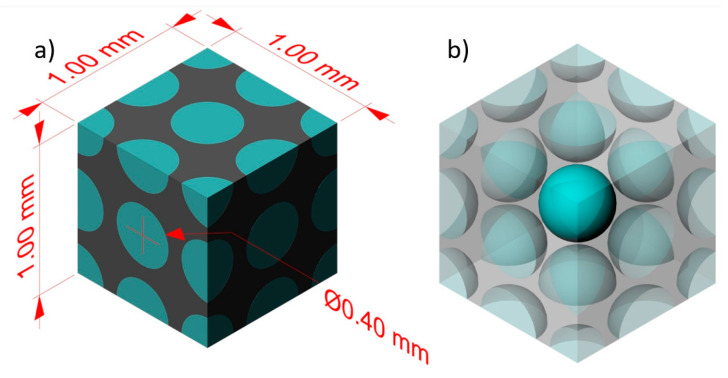
The geometry of NR foam used for the calculations: (**a**) external shaded model and (**b**) internal transparent model.

To investigate the effect of internal gas pressure on the mechanical properties of NR foams, a cube with dimensions of 1 × 1 × 1 mm^3^ was used. The cube contained a complete cell in its center, surrounded by a half cell and 4 quarter cells on each face (see Figure 1). In the limited literature about modeling polymer foams considering internal gas pressure, some researchers simplified the geometry to reduce calculations. They often modeled only a part of a complete unit cell. For instance, Xu et al. [32] used two geometric models: body-centered cubic (BCC) and face-centered cubic (FCC). In their geometry, they analyzed only one-eighth of the unit cell to account for symmetry and make the calculations more manageable. In this study, a complete cell was modeled in the center of the geometry (Figure 1), which interacted with half cells and quarter cells. The boundary conditions on the 6 faces of the cube are considered metric, so the behavior of these cells can be assumed to be similar to complete the internal foam cells. Through this geometry, the effect that cells have on each other (interactions) during the analysis can also be considered and will be the main advantage of this method.

### 2.2. Finite Element Model

**Figure 2:**
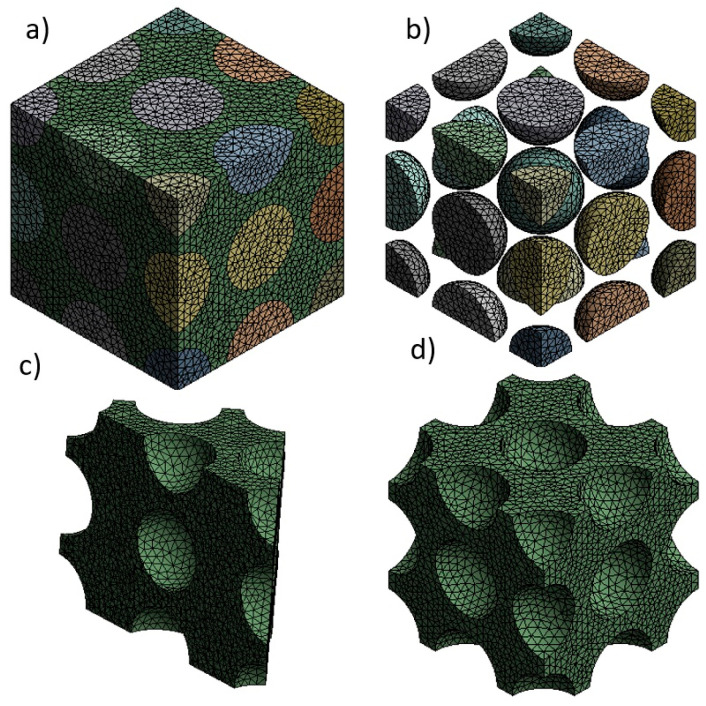
An example of the meshing used for the model with a cell size of 0.4 mm: (**a**) complete foam, (**b**) the array of cells, and (**c**,**d**) different views of the matrix.

A tetrahedral mesh was used in this study as it is an appropriate element to cover the spherical cells [35]. Figure 2 and Table 2 present the details of the meshing selected for the calculations.

Gaseous elements, which are defined as air material, were used to mesh the gas inside the cell, which is specially designed for modeling gases inside solids. Based on these conditions, the gas can be considered hydrostatic, i.e., the gas pressure is uniform throughout the internal cell surface. Each hydrostatic gas element overlaps a 3D solid element face around the cell. The model simulates the coupling between the gas volume and the surrounding solid matrix by applying the hydrostatic gas pressure as a surface load on the matrix. The system assumes no gas flow in or out of the matrix (impermeable boundary conditions).

When dealing with a coupled system of a natural rubber (matrix) surrounding a hydrostatic internal gas, the internal virtual work for the matrix needs to be augmented. This means that contributions from the gas must be considered. The internal energy expression can be used to start this process [36]:

(6) $$\overset{´}{W}=W+\int_{S_{s}}t_{si}v_{si}dS+\int_{S_{f}}t_{fi}v_{fi}dS$$

where *W* is the internal energy of the matrix, *S_s_* is the current matrix surface enclosing the cell volume, *S_f_* is the current gas surface enclosing the cell volume, *t_si_* is the component (*i*) of the surface traction at a point on *S_s_*, *t_fi_* is the component (*i*) of the surface traction at a point on *S_f_*, *v_si_* is the component (*i*) of velocity at a point on *S_s_,* and *v_fi_* is the component (*i*) of the velocity at a point on *S_f_*.

Equation (6) can be expanded as follows using the ideal gas law [36]:

(7) $${\dot{V}}_{f}=\frac{1}{∆t}\left(V_{f}-V_{nf}\right)=\frac{1}{∆t}\left(V_{f}-V_{f}\frac{P_{t}}{P_{nt}}\frac{T_{nt}}{T_{t}}\right)=-\frac{V_{f}}{V_{nt}}\dot{P}+\frac{V_{nf}}{V_{nt}}\dot{T}$$

where *Δt* is the time increment for the current sub-step, *V_nf_* is the gas volume at the end of the previous sub-step, *P_t_* (= *P_ref_* + *P*) is the total gas pressure, *P_ref_* is the reference pressure, *T_t_* (= *T_off_* + *T*) is the total temperature, *T_off_* is the temperature offset from absolute zero, *P_nt_* is the total fluid pressure at the end of the previous sub-step, and *T_nt_* is the total temperature at the end of the previous sub-step.

For compressible gas that does not follow the ideal gas law, the rate of change in volume can be expressed as [36]:

(8) $${\dot{V}}_{f}=\frac{1}{∆t}\left(V_{f}-V_{f}\frac{P_{t}}{P_{nt}}\frac{T_{nt}}{T_{t}}\right)=\frac{{∆V}_{f}}{∆P}\dot{P}+\frac{V_{nf}}{∆P}\dot{T}$$

where $\dot{P}$ is the slope (interpolated) of the pressure–volume data curve, and $\dot{T}$ is the slope (interpolated) of the temperature–volume data curve. The variation in the volume rate change (${\dot{V}}_{f}$) can be expressed as [36]:

(9) $${\dot{DV}}_{f}=\frac{{∆V}_{f}}{∆P}\dot{P}$$

where $\frac{{∆V}_{f}}{∆P}$ is the slope (interpolated) of the pressure–volume data curve, and $\dot{P}$ is the total gas pressure.

### 2.3. Assumptions and Limitations

**Figure 3:**
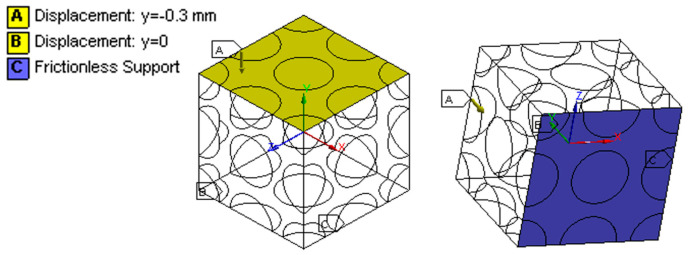
The boundary conditions of a representative volume element (RVE) from two viewpoints.

After the selection of a geometrical model as a representative volume element (RVE) for the NR foam, the upper surface was shifted by 0.3 mm along the Y axis to produce a 30% strain (to represent uniaxial compressive tests), while the lower surface was fixed (no displacement along the Y axis). A frictionless condition for the movement in the XZ plane was also imposed (Figure 3).

The 30% deformation limit was fixed due to limited time and resources for calculations and convergence. Another assumption is that the test strain rate does not affect the finite element method (FEM) solving. Although the parameter is important because the materials tested were viscoelastic, this parameter was not included in this study. So, to make the results consistent and reduce calculations, all the samples were deformed over one second (1 s).

This study investigated the effect of internal gas pressure on NR foams, with a focus on three main parameters: (1) the initial gas pressure within the cells (1, 2, and 3 atm), (2) the size of the cells’ diameters (D = 0.1, 0.2, 0.3, and 0.4 mm), and (3) how the foam behavior changed at the micro level when connecting holes were present between the closed cells with different opening sizes (hole diameter size, d = 0.07, 0.08, 0.09, and 0.1 mm). In all cases, the gas pressure was the absolute pressure, and the gas was assumed to be a hydrostatic fluid, i.e., the pressure was uniform throughout the cell (inside the surface of cells). The system was closed as no gas flow in or out of the matrix occurred. Finally, the increased hydrostatic pressure of the gas was only related to volume changes.

It is necessary to mention that a fourth condition was added to investigate the effect of the initial internal gas pressure, called “No Gas”. For this condition, it is assumed that the third part of Equation (1) related to the gas modulus ($E_{gas}$) is disregarded, so the mechanical behavior of the foam is obtained by Young’s modulus of the edges ($E_{edge}$) and Young’s modulus of the surface ($E_{surface}$). From a physical point of view, this model can be described as if there was no absolute pressure change inside the cell during deformation, i.e., the internal and external pressures are equal. Consequently, no gas volume or internal gas pressure changes were observed, and the results were used for comparison purposes.

A dimensionless value was introduced to express changes in the internal gas pressure by dividing the internal gas pressure (P) by the initial gas pressure (P_0_) as:

(10) $$\alpha =\left(\frac{P}{P_{0}}\right)$$

The ideal gas hypothesis was also used to relate the internal gas pressure and volume change in the cells. According to Boyle’s law, the relation is:

P V = n R T(11)

where V is the cell volume, n is the number of gas moles, R is the ideal gas constant, and T is the absolute temperature. So, Boyle’s law states that at a constant temperature, the product between pressure and volume is constant. Mathematically, this can be expressed as:

P_0_ V_0_ = P_1_ V_1_ (12)

where P_0_ and V_0_ are the initial gas pressure and initial cell volume, while P_1_ and V_1_ are the secondary gas pressure and secondary cell volume.

In order to perform a more quantitative analysis, the root-mean-square error (RMSE) of all samples was calculated with respect to a single sample to quantify the changes obtained. The RMSE was calculated as:

(13) $$RMSE=\sqrt{\frac{\sum_{i=1}^{N}{\left(x_{i}-y_{i}\right)}^{2}}{N}}$$

where *N* is the number of non-missing data points, $x_{i}$ is the value of the first sample, and $y_{i}$ is the value of a second sample.

In order to complete the analyses and determine the sensitivity of each effect, the rate of variation (increase or decrease) in each parameter was calculated as follows:

(14) $$Rate\;of\;change=\frac{y_{max}-y_{min}}{x_{max}-x_{min}}$$

Also, for each result obtained from FEMs, an attempt was made to provide a mathematical model using regression methods to predict the variables of interest. To assess the goodness of fit of the models, the coefficient of determination R^2^ was calculated as follows:

(15) RSS=∑i=1N(Pi−P^i)2,TSS=∑i=1N(Pi−P¯i)2,R2=1−RSSTSS

where $P_{i}$ represents the FEM values and ${\hat{P}}_{i}$ are the model-fitted values, while ${\bar{P}}_{i}$ represents the mean of the FEM values, and *N* represents the number of FEM data points used in the fit.

## 3. Results and Discussion

As described in the introduction, this study is divided into three main categories: (1) initial gas pressure, (2) initial cell size, and (3) the presence of connecting holes between the cells. In the discussion, the results are presented and analyzed for each effect. It should be noted that each section is also divided into two sub-sections: (1) results for uniaxial compression and (2) discussion/comparison. An attempt was made to present the results quantitatively before being examined in more detail for trends.

### 3.1. Effect of Initial Gas Pressure

Three samples based on the geometry presented in Figure 1 with varying initial internal gas pressures (1, 2, and 3 atm) were studied, as well as one sample without initial gas pressure. The samples were subjected to uniaxial compression up to 30% strain to study the relationships between stress and strain.

#### 3.1.1. Uniaxial Compression Results

**Figure 4:**
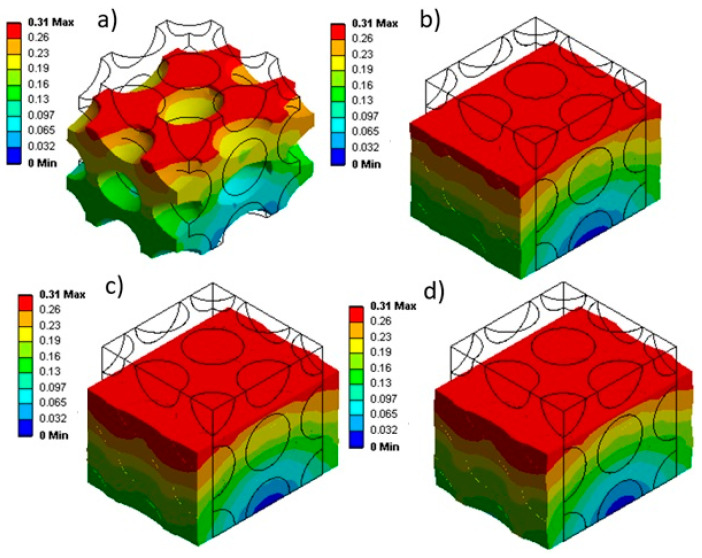
Displacement plots under uniaxial compression: (**a**) without internal gas pressure and with different initial internal gas pressure: (**b**) 1 atm, (**c**) 2 atm, and (**d**) 3 atm.

Upon solving the FEM, the results for the deformation are presented in Figure 4. It can be seen that while the lower (bottom) surface of the sample remains immobile (indicated in blue), the upper (top) surface experiences a displacement of 0.3 mm (30% strain) due to the external axial load applied (highlighted in red). It can be seen that a complex (non-uniform) distribution across thickness is generated depending on the foam structure.

**Figure 5:**
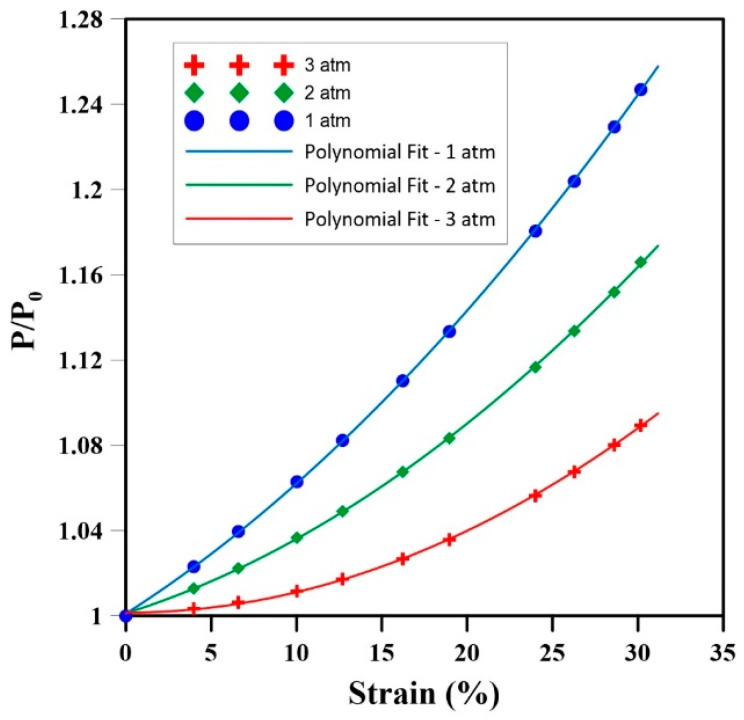
Relative internal gas pressure as a function of deformation under uniaxial compression.

Based on the deformation results from the FEM, Figure 5 depicts how the internal gas pressure evolves for the samples with different initial gas pressures (1, 2, and 3 atm) inside the cells. The main results are compiled in Table 3, including the statistical values (RMSE and rate) of internal gas pressure increase. Based on these results, a second-order polynomial regression is proposed to represent the dimensionless relationship between the internal gas pressure changes (*α*) and strain (ϵ) as:

(16) $$\alpha =\left(\frac{P}{P_{0}}\right)=A_{0}+A_{1}\epsilon +A_{2}\epsilon^{2}$$

where *A*_0, 1, 2_ are model parameters. The coefficients of determination (R^2^) are all above 0.99, indicating good fitting under the conditions investigated.

The results of Table 3 show that the RMSE for an initial gas pressure of 3 atm compared to 1 atm is the lowest but 92% lower than a sample starting at 2 atm. Additionally, the samples with lower initial cell pressure show a higher rate of change in α. For example, the rate at 1 atm is 158% higher than for 3 atm.

**Figure 6:**
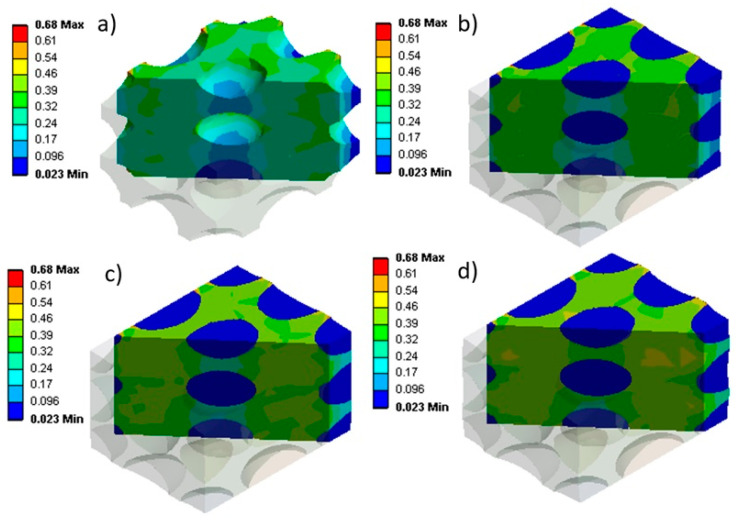
Stress (MPa) during uniaxial compression for different initial internal gas pressures: (**a**) without internal gas pressure, (**b**) 1 atm, (**c**) 2 atm, and (**d**) 3 atm.

Considering the initial gas pressure and changes in internal gas pressure during uniaxial compression, Figure 6 presents the stress distribution for each sample. As expected, a sample with a lower initial gas pressure has lower stress levels, indicated by a light blue contour within the material. But by increasing the initial gas pressure from 1 atm to 3 atm, the color contour transitions from green to yellow, indicating higher local stress levels. Figure 6a illustrates the stress state of the sample without considering the internal gas pressure. This condition generates the lowest stress levels as no resistance is generated by the internal gas pressure inside the cells.

**Figure 7:**
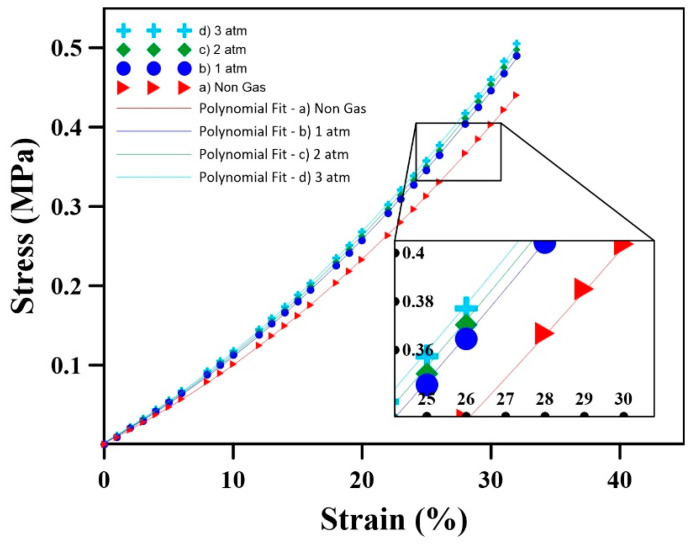
Stress as a function of strain for different initial internal gas pressures through uniaxial compression.

The results of the stress (*σ*) and strain (ϵ) obtained for the samples analyzed are reported in Figure 7, while the main values are presented in Table 4. This Table also reports the parameters of a second-order polynomial regression model as follows:

(17) $$\sigma =B_{0}+B_{1}\epsilon +B_{2}\epsilon^{2}$$

where *B*_0, 1, 2_ is the model parameter for uniaxial compression. The coefficients of determination (R^2^) are all above 0.95, indicating good fitting under the conditions investigated.

Figure 7 shows that increasing the initial gas pressure from 1 atm to 3 atm increases the stress levels. For example, the RMSE at 3 atm compared to 1 atm is 65% higher than the RMSE at 2 atm compared to 1 atm. Also, the rate of stress increase is higher for samples with higher initial internal gas pressure. For instance, the rate for 3 atm is 3% higher than for 1 atm (Table 4).

#### 3.1.2. Discussion on the Effect of Initial Gas Pressure

The results of Figure 5, Figure 6 and Figure 7 clearly show how the initial gas pressure significantly influences the mechanical response of NR foams during uniaxial compression. It can be seen that higher initial gas pressure inside the cells decreases the changes associated with the relative internal gas pressure (α) and increases the stress levels during compression. This aligns with fundamental gas behavior principles as higher initial gas pressures resist more external stresses when applied, and the rate of increasing internal gas pressure is lower when compressed.

The higher relative Internal gas pressure (α) values at lower initial gas pressures for a specific strain can be rationalized using Boyle’s law (Equation (11)) and the NR foam’s resistance to external uniaxial forces. During compression, the internal force of the sample opposes the external axial force because of the cell’s walls and the internal gas pressure. At a higher initial gas pressure, the material’s ability to resist compression increases due to the internal gas pressure. According to Boyle’s law, the presence of this higher gas pressure inside the cell causes smaller volume changes in the cells. In contrast, a lower initial gas pressure leads to larger volume changes and higher α values. This is because the volume can compress more, resulting in higher internal gas pressure changes and easier external force and deformation.

The stress–strain behavior, as depicted in Figure 5 and Figure 7, illustrates a clear correlation between the initial gas pressure and stress levels. Higher initial gas pressure increases the internal resistance to compression, consequently increasing the stress levels within the sample, especially at a higher initial gas pressure for a fixed strain (Figure 6). This increased gas pressure increases the stiffness of the sample, resulting in a more pronounced stress response during compression. These findings underscore the importance of internal gas pressure on the mechanical behavior of rubber foams, revealing that P_0_ significantly affects the material’s response to compression. Contrary to some of the literature neglecting the role of internal gas pressure [21], these results emphasize its substantial effect on the stress levels in rubber foams subjected to compression.

Finally, a mathematical model (second-order polynomial) regression is proposed to predict the relationship between strain, stress, and initial internal gas pressure as:

(18) $$\sigma =C_{0}+C_{1}P_{0}+C_{2}{P_{0}}^{2}+C_{3}\epsilon +C_{4}\epsilon^{2}+C_{5}P_{0}\epsilon$$

where *C*_0, 1, 2, 3, 4, 5_ is the model parameter, as reported in Table 5. Again, good fitting is obtained with R^2^ > 0.99.

These findings support the idea that the initial gas pressure plays a pivotal role in determining the mechanical behavior of rubber foams under compression, as reported elsewhere [4,8,34]. Equation (18) can be used to predict the compression properties of rubber foams for specific applications. Furthermore, the correlation can be used to optimize and design/engineer new NR foams with tailored mechanical properties, offering a potential pathway for important advancements in polymer engineering and science.

### 3.2. Effect of Cell Size

The effect of initial cell size (D) on the variation in internal gas pressure under compression is investigated next. Again, four samples are considered based on the geometries presented in Figure 1. As a first step, the initial internal gas pressure was set at P_0_ = 1 atm and the diameter of initial cell sizes at D = 0.1, 0.2, 0.3, and 0.4 mm. For easy comparison, each sample underwent uniaxial compression tests, as in the previous section.

#### 3.2.1. Uniaxial Compression Results

**Figure 8:**
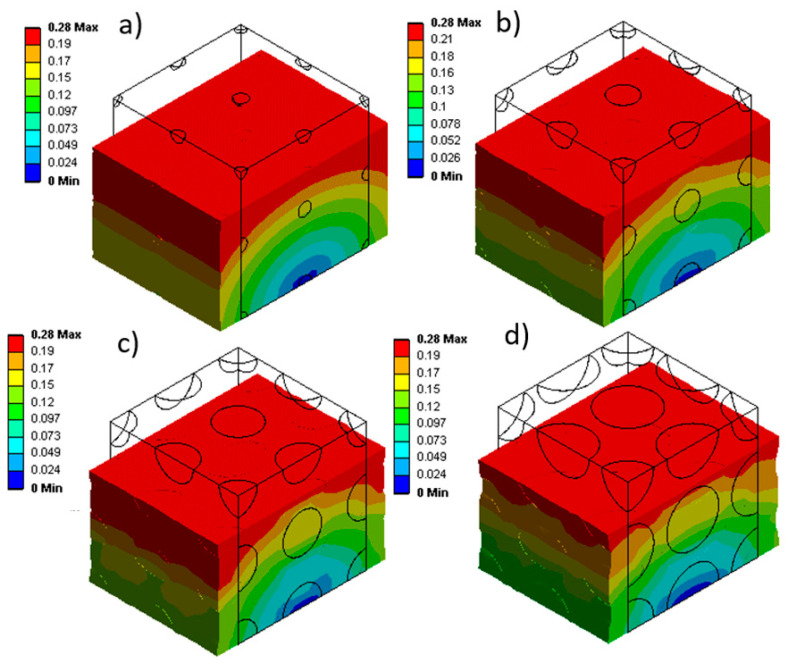
Displacement (mm) under uniaxial compression for different initial cell sizes with diameters (D) of (**a**) 0.1 mm, (**b**) 0.2 mm, (**c**) 0.3 mm, and (**d**) 0.4 mm (P_0_ = 1 atm).

After solving the FEM, the displacement profiles are presented in Figure 8. The uniaxial compression test subjected all four samples with different cell sizes (D = 0.1, 0.2, 0.3, and 0.4 mm) to compression. The lower surface (highlighted in blue) remained fixed, while the upper surface (highlighted in red) was axially shifted to achieve a 30% strain.

**Figure 9:**
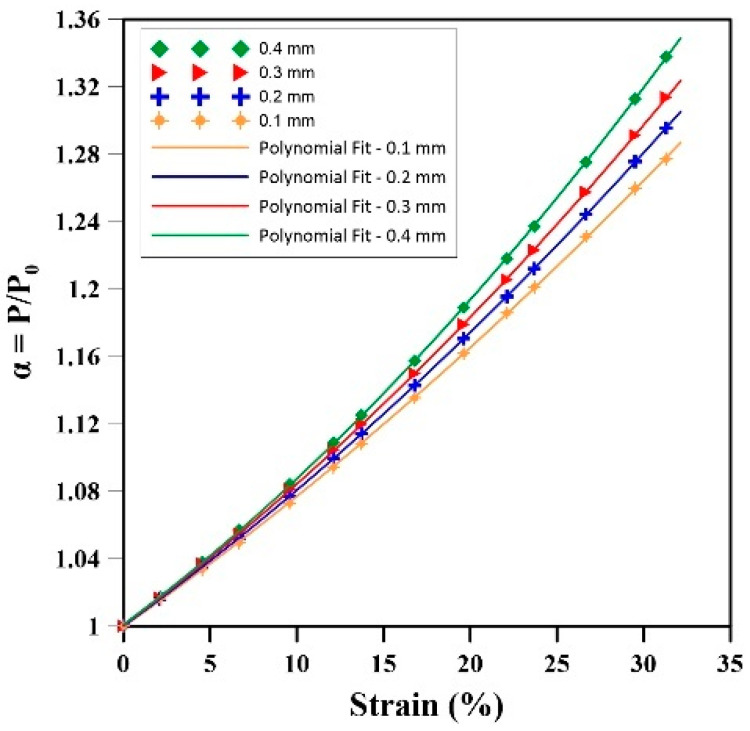
Relative internal gas pressure as a function of deformation for different cell sizes during uniaxial compression.

After solving the FEM problem, Figure 9 presents the results of the change in relative internal gas pressure (α) for the samples under uniaxial compression test, while Table 6 reports the statistical values (RMSE and rate) of these results. Based on these values, the curves were fitted to a second-order polynomial regression as:

(19) $$\alpha =\left(\frac{P}{P_{0}}\right)=D_{0}+D_{1}\epsilon +D_{2}\epsilon^{2}$$

where *D*_0, 1, 2_ are the model parameters, and their values are reported in Table 6. The fittings are good since R^2^ > 0.95.

The results of Figure 9 and Table 6 show a clear relationship between strain, cell size, and changes in internal gas pressure. With increasing strain, larger cell sizes exhibit significantly higher increases in internal gas pressure than smaller cell sizes. For example, a cell size of 0.4 mm exhibits the highest RMSE (3.14 × 10^−2^ atm) for changes in internal gas pressure. This value represents an increase of 227% and 63% compared to samples having D = 0.2 mm and 0.3 mm, respectively (Table 6). Moreover, the rate of gas pressure increase inside the cells was notably higher for samples with larger cell sizes. For instance, the rate of pressure changes increases by 22% as the cell size increases from 0.1 mm to 0.4 mm. These results suggest that larger cell sizes are more effective in accommodating and retaining gas, leading to a more substantial pressure increase during compression. Their higher rate of pressure increase can be attributed to a larger volume available for gas retention and more deformability/elasticity.

**Figure 10:**
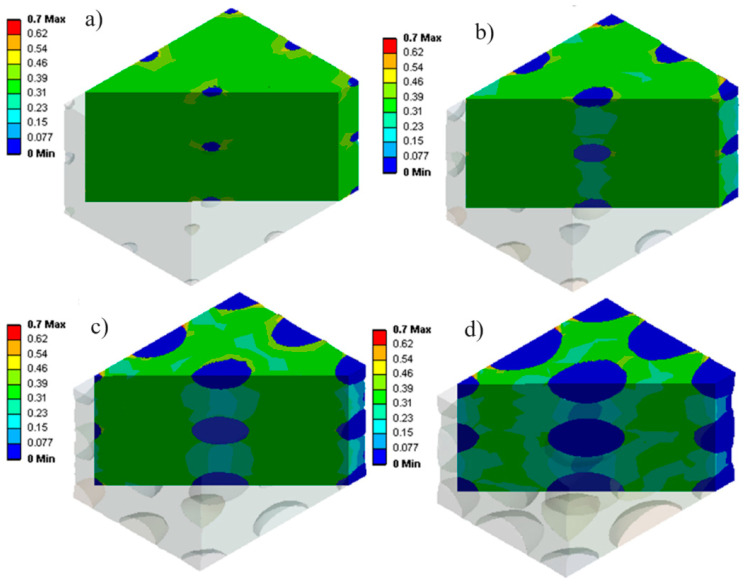
Stress (MPa) distribution during uniaxial compression for different cell sizes (D) of (**a**) 0.1 mm, (**b**) 0.2 mm, (**c**) 0.3 mm, and (**d**) 0.4 mm.

Considering the changes in internal gas pressure (Figure 9), the stress distribution in the materials was determined to analyze their mechanical behavior. Figure 10 shows that for larger cells (0.4 mm in Figure 10a), more non-uniformity is created in the stress distribution inside the matrix (light blue, dark blue, and green). However, as the cell sizes become smaller (down to 0.1 mm in Figure 10d), the stress distribution becomes more homogeneous (almost green color everywhere). As reported in the literature [4,8,34], smaller cells seem to be better, especially in terms of uniformity and distribution of mechanical stresses.

Figure 10 also shows that stress concentration (yellow) around the cells occurs due to a higher concentration of internal gas pressure. In particular, Figure 10 clearly illustrates the distinction between the stress distribution inside the cells and inside the rubber matrix.

**Figure 11:**
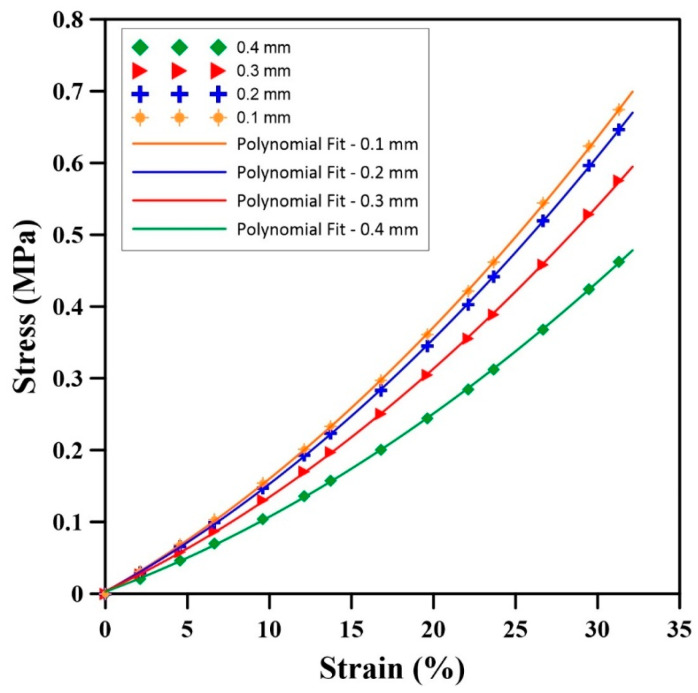
Stress as a function of strain for different cell sizes during uniaxial compression.

Figure 11 presents the stress–strain plots for the samples with different cell sizes subjected to uniaxial compression. Again, an attempt was made to obtain an equation to represent the finite element results for the relationship between stress and strain:

(20) $$\sigma =E_{0}+E_{1}\epsilon +E_{2}\epsilon^{2}$$

where $E_{0,\;1,\;2\;}$ are the model parameters under uniaxial compression, and Table 7 reports their values. These curves are well fitted for the different diameters as R^2^ > 0.95.

Figure 11 and Table 7 show that increasing the cell size from 0.1 mm to 0.4 mm decreases the stress values at constant strains. For instance, the RMSE at 0.1 mm compared to 0.4 mm is 15% higher than for D = 0.2 mm. Also, the rate of stress increase is higher for samples having smaller cell sizes. For instance, the increase rate of stress at D = 0.1 mm is 45% higher than for D = 0.4 mm.

#### 3.2.2. Discussion on the Effect of Cell Size

The results clearly show the pivotal role of cell sizes and changes in internal gas pressure within natural rubber foams during uniaxial compression. A direct link between cell dimensions and changes in internal gas pressure was observed in Figure 9, where larger cell sizes displayed a greater tendency for increased internal gas pressure with strain. Based on Boyle’s law (Equation (11)), larger cell sizes, for a fixed initial gas pressure, have a greater ability to change volume (more compressibility). Also, these large cells participate earlier and more significantly during loading in the volume change in the whole sample. As the sample is subjected to uniaxial compression, a larger volume at constant P_0_ allows for more volume decreases, leading to higher relative internal gas pressure increases.

To discuss the stress changes inside the samples, Figure 10 shows that the variation in cell sizes increases (more blue and green zones) with deformation. This issue can be caused by the resistance of the material against the external force. In this way, smaller cells show more resistance against loading conditions, thus increasing the average internal stresses in the cell (more green zones). However, due to the concentration of stress inside the cell around small cells, the change in stress in the matrix around the cell is more limited.

The stress curve analysis in Figure 11 revealed an inverse relationship between cell sizes and stress levels in the samples subjected to compression. Smaller cell sizes (D = 0.1 mm) exhibited higher stress values compared to larger cell sizes (D = 0.4 mm). This is due to a more restricted gas volume within smaller cells, limiting space for deformation, coupled with the effect of internal gas pressure, which amplifies the stress level during compression. As a result, smaller cells exhibit reduced deformation capacity, leading to higher stress and increased stiffness to deformation. This illustrates the significant effect of cell size on the stress distribution inside NR foams under compression.

Finally, using the results presented in this section, a second-order polynomial is proposed to determine a relationship between the stress, the strain, and the cell size:

(21) $$\sigma =F_{0}+F_{1}D+F_{2}D^{2}+F_{3}\epsilon +F_{4}\epsilon^{2}+F_{5}D\epsilon$$

where $F_{0,\;1,\;2,\;3,\;4,\;5}$ are the model parameters, and their values are reported in Table 8. Again, good fitting is obtained with R^2^ > 0.99.

This part emphasized the significant effect of cell sizes on the mechanical behavior of NR foams. Understanding the relationships between cell size, internal gas pressure, compression stress, and strain can lead to better foam design with tailored properties over a wide range of industrial applications, especially for closed-cell foams. The next section presents some results when some defects (cell opening) are present.

### 3.3. Effect of Cell Connection on Internal Gas Pressure

The interconnected arrangement of cells in NR foams often leads to the formation of holes at their intersections, resulting in local stress concentration and hot spots that can potentially cause structural damage, such as coalescence and break-up. In order to explore this effect, a model consisting of two interconnected cells with varying hole diameters (d = 0.07, 0.08, 0.09, and 0.1 mm) is considered. The geometry in this section is cubic and similar to Figure 1, but only two cells are connected horizontally in their center. The diameter of the cells is 0.4 mm, and they are placed symmetrically in the center of the cube. The boundary conditions are similar to those in Figure 3, and the sample is again subjected to uniaxial compression in the vertical direction compared to the cells.

#### 3.3.1. Uniaxial Compression Results

**Figure 12:**
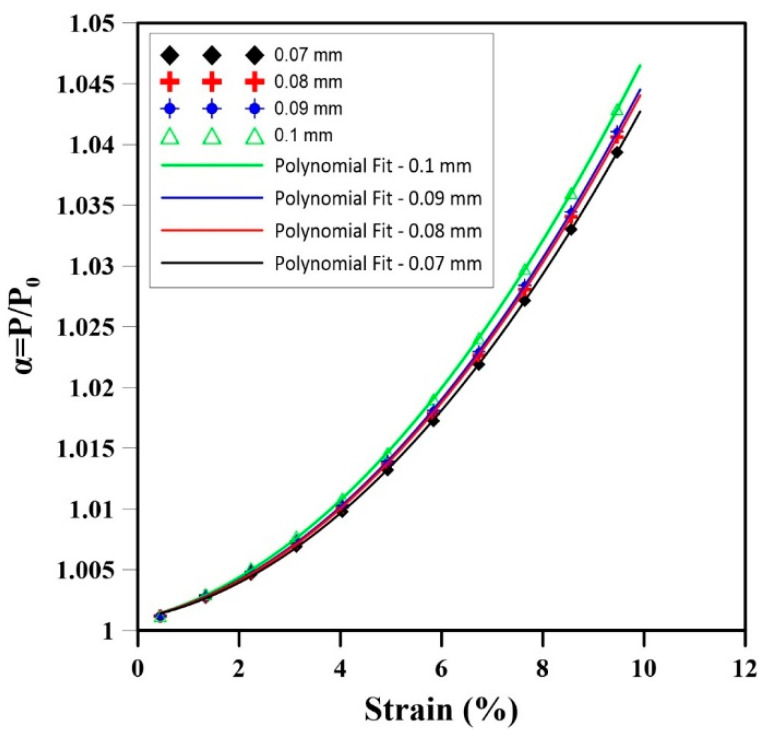
Relative internal gas pressure as a function of strain for different interconnected cells’ hole diameters during uniaxial compression.

The uniaxial compression tests on NR foam samples with interconnected cells highlighted an important relationship between the hole size (cell intersections) and the subsequent change in internal gas pressure. Four samples with hole diameters of 0.07, 0.08, 0.09, and 0.1 mm were analyzed, and the results are presented in Figure 12 and Table 9. As the strain increased, the internal gas pressure also increased in all samples, but samples with larger hole sizes showed a more substantial elevation in internal gas pressure. As reported in Table 9, the difference in RMSE of the sample with a hole connection size of 0.07 mm compared to 0.1 mm is the highest (2.0 × 10^−3^), which is 92% less than for a hole of 0.09 mm. Additionally, the rate of increase in gas pressure within the cells was notably higher in samples with larger hole sizes. The rate of change in the internal gas pressure decreases by 8% by reducing the hole connection size from 0.1 mm to 0.07 mm. Based on these findings, a second-order polynomial regression model is proposed as:

(22) $$\alpha =\left(\frac{P}{P_{0}}\right)=G_{0}+G_{1}\epsilon +G_{2}\epsilon^{2}$$

where *G*_0,1,2_ is the model parameter, and ϵ is the strain during the uniaxial compression test. Good fitting is again obtained with all R^2^ > 0.95.

**Figure 13:**
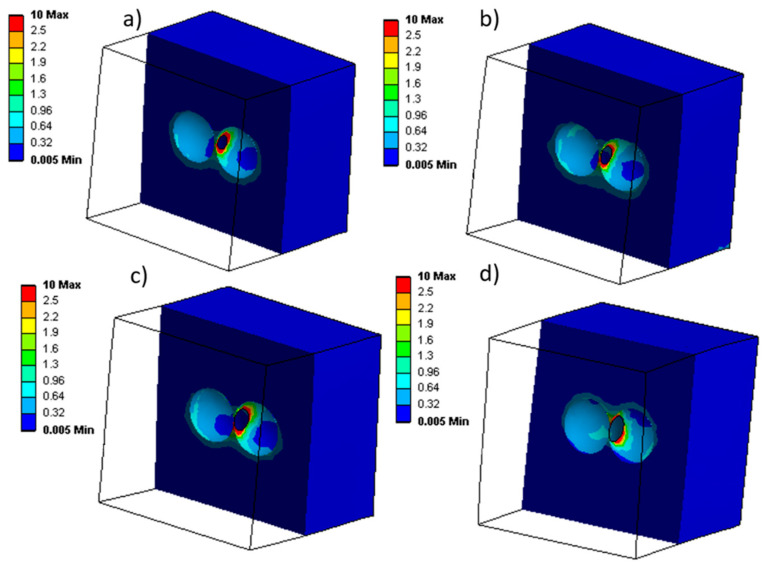
Local stress concentration during uniaxial compression with different connecting hole diameters: (**a**) 0.07 mm, (**b**) 0.08 mm, (**c**) 0.09 mm, and (**d**) 0.1 mm.

**Figure 14:**
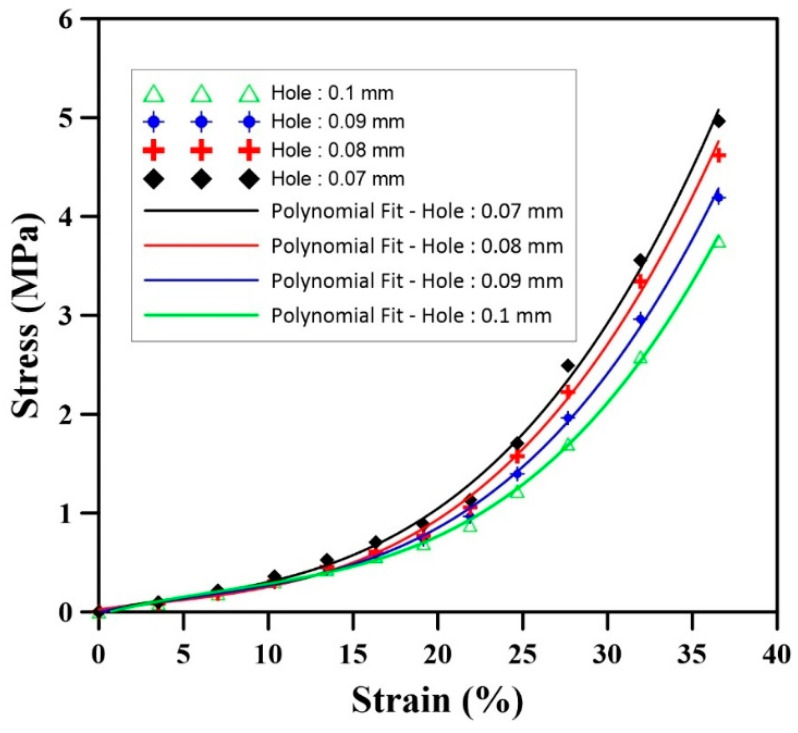
Stress–strain curve for different connecting hole sizes during uniaxial compression.

When assessing the stress distribution during the uniaxial compression test, the local stresses are shown in Figure 13. Figure 13 shows that the stress is concentrated around the holes during uniaxial compression. A careful analysis of the results shows that increasing the hole diameter from 0.07 to 0.1 mm decreases the level of local stress concentrated around the hole (less red color contour). Based on Figure 14, the stress–strain plot of these results show that during compression and for a constant strain, a higher stress value is generated for samples with smaller holes. The statistical values are presented in Table 10. Based on these results, the RMSE of the smallest hole size (0.07 mm) compared to the largest (0.1 mm) is 5.1 × 10^−1^ MPa, which is 173% higher than the RMSE for a 0.09 mm hole. Also, the rate of stress increase is higher for samples with smaller hole sizes. For instance, the increasing rate at 0.07 mm is 32% higher than at 0.1 mm (Table 10).

Based on these findings, a third-order polynomial was selected to obtain a good regression model (R^2^ > 0.95) as:

(23) $$\sigma =H_{0}+H_{1}\epsilon +H_{2}\epsilon^{2}+H_{3}\epsilon^{3}$$

where *H*_0,1,2,3_ is the model parameter as reported in Table 10.

#### 3.3.2. Discussion on the Effect of Cell Connection

The results of this study showed that the internal gas pressure inside the cells of natural rubber foams has a significant effect on the mechanical properties in compression (Figure 12). As the strain increased, the internal gas pressure increased in all samples, but samples with larger connecting hole sizes exhibited a higher increase in internal gas pressure. This is likely due to the fact that larger holes allow for more gas to flow between the cells, resulting in an easier volume change in the sample subjected to uniaxial compression, leading to higher internal gas pressure.

The stress concentration around the holes was also found to be affected by the size of the holes (Figure 13). As the hole diameter increased, the level of local stress concentration decreased (Figure 14). This is likely due to the fact that larger holes allow for the stress to be more easily distributed around the hole (larger diameter = larger circumference/surface, rather than being concentrated in a smaller area (small diameter = small circumference/surface).

These results have important implications for the design of natural rubber foams. By controlling the size and number of holes in the cells, it is possible to tailor the mechanical properties of the material to meet specific requirements. For example, if a material with high strength is required, then cells with small holes should be used. Conversely, if a material with high compliance is required, then cells with larger holes should be used.

The results of this study also provide insights into the failure mechanisms of natural rubber foams. Stress concentration around the holes is a potential failure site. It is also more likely that materials with larger holes will be more susceptible to failure at this point. This suggests that it is important to carefully consider the size and distribution of holes when designing natural rubber foams for critical applications.

Based on all the conditions investigated in this section, a model has been presented using a third-order polynomial:

(24) $$\sigma (d,\epsilon )=\beta_{0}+\beta_{1}d+\beta_{2}\epsilon +\beta_{3}d^{2}+\beta_{4}d\epsilon +\beta_{5}\epsilon^{2}+\beta_{6}d^{3}+\beta_{7}d^{2}\epsilon +\beta_{8}d\epsilon^{2}+\beta_{9}\epsilon^{3}$$

where *β*_0,1,2,3,4,5,6,7,8,9_ is the model parameter reported in Table 11, *d* is the hole diameter, σ is the stress, and ϵ is the strain subjected to uniaxial compression. Good fitting is obtained with R^2^ > 0.99.

In summary, these findings emphasize the importance of hole size at cell connections on both the internal gas pressure changes and stress distribution in rubber foams subjected to mechanical compression. Understanding these relationships offers valuable knowledge to optimize the foam structure and mitigate potential structural weaknesses, leading to improved overall mechanical properties.

## 4. Conclusions

This comprehensive study investigated the effect of internal gas pressure and cell characteristics (cell size and connecting hole) on the mechanical behavior of closed-cell natural rubber foams subjected to compression. The main conclusions are:

1. Effect of Internal Gas Pressure and Initial Gas Pressure:
  - Higher initial gas pressure led to lower internal gas pressure changes and higher stress levels during compression, leading to a significantly increased mechanical resistance.
  - For example, the rate of stress increase was 3% higher for samples with an initial gas pressure of 3 atm compared to those starting at 1 atm, while the RMSE was 65% higher.
2. Effect of Cell Size Considering Internal Gas Pressure:
  - Larger cell sizes displayed a substantial increase in internal gas pressure during compression, resulting in higher stress levels, while smaller cell sizes exhibited increased stress concentration and resistance to deformation.
  - For example, the RMSE of samples with a cell size of 0.1 mm was 15% higher, with a 45% higher rate of stress increase compared to 0.4 mm.
3. Effect of Internal Gas Pressure on Cell Connection:
  - Larger hole sizes led to a higher increase in internal gas pressure and stress.
  - For instance, the RMSE of samples with a hole size of 0.07 mm was 173% higher, with a 32% higher rate of stress increase compared to 1.0 mm.

This research indicates a vital link between internal gas pressure, cell characteristics, and the resulting mechanical response of rubber foams. These quantitative results highlighted the effect of initial gas pressure, cell size, and hole size on stress levels and deformation resistance. The proposed correlations based on polynomial regressions provided predictive tools to quantitively understand the relationships between strain, stress, initial gas pressure, cell size, and hole size, enabling potential engineering applications to control/optimize the mechanical behavior of rubber foams under compression. These findings set the stage for improved material design strategies and application development in polymer engineering as they are expected to apply to other matrices and types of solicitation for a more general understanding of polymer foam behavior.

## Figures and Tables

**Table 1:** Material constants for the Mooney-Rivlin 5 parameter model (Equation (5)).

Parameter	Value [MPa]
C_01_	0.059
C_02_	0.128
C_10_	0.397
C_11_	1.36
C_20_	3.78

**Table 2:** Meshing information for the models used.

Cell Size (mm)	# Nodes	# Elements
0.1	191,378	132,965
0.2	192,514	134,932
0.3	196,979	135,556
0.4	204,516	137,172

**Table 3:** Statistical values and model parameters (Equation (16)) for the relative internal gas pressure.

Initial Internal Gas Pressure (atm)	1	2	3
*A*_0_	1.0009	1.0011	1.0014
*A*_1_	5.10 × 10^−3^	2.53 × 10^−3^	−5.23 × 10^−7^
*A*_2_	1.00 × 10^−4^	9.63 × 10^−5^	9.62 × 10^−5^
R^2^	0.99996	0.99991	0.99973
Rate of α	7.8 × 10^−3^	5.3 × 10^−3^	3.0 × 10^−3^
RMSE of the internal gas pressure compared to P_0_ = 1 atm	-	5.2 × 10^−2^	1.0 × 10^−1^

**Table 4:** Statistical values and model parameters (Equation (17)) for the stress and strain relation using different initial internal gas pressures.

Initial Internal Gas Pressure (atm)	1	2	3	No Gas
*B*_0_	2.19 × 10^−3^	2.57 × 10^−3^	2.63 × 10^−3^	1.92 × 10^−3^
*B*_1_	8.94 × 10^−3^	9.15 × 10^−3^	9.46 × 10^−3^	8.08 × 10^−3^
*B*_2_	1.94 × 10^−4^	1.95 × 10^−4^	1.93 × 10^−4^	1.76 × 10^−4^
R^2^	0.99991	0.99991	0.99992	0.99996
Rate of stress (MPa)	1.96 × 10^−2^	1.99 × 10^−2^	2.02 × 10^−2^	1.76 × 10^−2^
RMSE of the stress compared to P_0_ = 1 atm	-	4.6 × 10^−3^	9.3 × 10^−3^	2.5 × 10^−2^

**Table 5:** Fitting parameters (Equation (18)) for the combined effect of strain and initial internal gas pressure on compression stress (MPa).

*C*_0_	*C*_1_	*C*_2_	*C*_3_	*C*_4_	*C*_5_	R^2^
−9.22 × 10^−4^	1.16 × 10^−2^	−4.06 × 10^−3^	8.01 × 10^−3^	1.89 × 10^−4^	5.96 × 10^−4^	0.9993

**Table 6:** Statistical values and model parameters (Equation (19)) of the relative internal gas pressure changes for different cell sizes.

Initial Cell Diameter	0.1 mm	0.2 mm	0.3 mm	0.4 mm
*D*_0_	0.9997	1.0001	1.0005	1.0009
*D*_1_	7.19 × 10^−3^	7.41 × 10^−3^	7.62 × 10^−3^	7.67 × 10^−3^
*D*_2_	5.43 × 10^−5^	6.49 × 10^−5^	7.57 × 10^−5^	9.83 × 10^−5^
R^2^	0.99994	0.99999	0.99999	0.99998
Rate of α	1.15 × 10^−2^	1.23 × 10^−2^	1.30 × 10^−2^	1.41 × 10^−2^
RMSE of α compared to D = 0.1 mm	-	9.60 × 10^−3^	1.92 × 10^−2^	3.14 × 10^−2^

**Table 7:** Statistical values and model parameters (Equation (20)) of the stress–strain curve for different cell sizes.

Initial Cell Diameter	0.1 mm	0.2 mm	0.3 mm	0.4 mm
*E*_0_	2.69 × 10^−3^	3.00 × 10^−3^	3.62 × 10^−3^	2.84 × 10^−3^
*E*_1_	1.31 × 10^−2^	1.24 × 10^−2^	1.07 × 10^−2^	8.47 × 10^−3^
*E*_2_	2.68 × 10^−4^	2.60 × 10^−4^	2.40 × 10^−4^	1.97 × 10^−4^
R^2^	0.99995	0.99995	0.99991	0.99991
Rate of stress (MPa)	2.7 × 10^−2^	2.6 × 10^−2^	2.3 × 10^−2^	1.9 × 10^−2^
RMSE of the stress compared to D = 0.4 mm	1.2 × 10^−1^	1.0 × 10^−1^	6.3 × 10^−2^	-

**Table 8:** Statistical values and model parameters (Equation (21)) of the stress–strain curve for different cell hole connection sizes.

*F*_0_	*F*_1_	*F*_2_	*F*_3_	*F*_4_	*F*_5_	R^2^
−5.57 × 10^−2^	5.27 × 10^−1^	−9.74 × 10^−1^	1.69 × 10^−2^	2.41 × 10^−4^	−2.31 × 10^−2^	0.9986

**Table 9:** Statistical values and model parameters (Equation (22)) of the stress–strain curve for different connecting hole cell sizes.

Connecting Hole Size	0.07 mm	0.08 mm	0.09 mm	0.1 mm
*G*_0_	1.00098	1.00097	1.00097	1.00094
*G*_1_	7.95 × 10^−4^	8.64 × 10^−4^	8.98 × 10^−4^	9.94 × 10^−4^
*G*_2_	3.44 × 10^−4^	3.50 × 10^−4^	3.52 × 10^−4^	3.63 × 10^−4^
R^2^	0.99995	0.99995	0.99995	0.99995
Rate of internal gas pressure	4.5 × 10^−3^	4.65 × 10^−3^	4.70 × 10^−3^	4.9 × 10^−3^
RMSE of the stress compared to d = 0.1 mm	2.0 × 10^−3^	1.3 × 10^−3^	1.0 × 10^−3^	-

**Table 10:** Statistical values and model parameters (Equation (23)) of the stress–strain curve for different connecting hole cell sizes.

Connecting Hole Size	0.07 mm	0.08 mm	0.09 mm	0.1 mm
*H*_0_	2.02 × 10^−2^	2.57 × 10^−2^	−8.17 × 10^−4^	−2.74 × 10^−2^
*H*_1_	2.75 × 10^−2^	2.12 × 10^−2^	3.38 × 10^−2^	4.63 × 10^−2^
*H_2_*	−1.03 × 10^−3^	−8.90 × 10^−4^	−1.78 × 10^−3^	−2.66 × 10^−3^
*H*_3_	1.11 × 10^−4^	1.05 × 10^−4^	1.11 × 10^−4^	1.17 × 10^−4^
R^2^	0.99691	0.99737	0.99838	0.99905
Rate of stress increase (MPa)	1.4 × 10^−1^	1.3 × 10^−1^	1.2 × 10^−1^	1.1 × 10^−1^
RMSE of the stress compared to d = 0.1 mm	5.1 × 10^−1^	3.7 × 10^−1^	1.9 × 10^−1^	-

**Table 11:** Statistical values and model parameters (Equation (24)) of the stress–strain curve for different cell hole connection sizes.

$\beta_{0}$	$\beta_{1}$	$\beta_{2}$	$\beta_{3}$	$\beta_{4}$	$\beta_{5}$	$\beta_{6}$	$\beta_{7}$	$\beta_{8}$	$\beta_{9}$	R^2^
1.24	−25.29	−9.24 × 10^−2^	87.49	2.45	2.30 × 10^−3^	420.62	11.36	−4.57 × 10^−2^	1.11 × 10^−4^	0.9976

## Data Availability

The raw data supporting the conclusions of this article will be made available by the authors on request.
